# Validation of the Vascular quality of life questionnaire – 6 for clinical use in patients with lower limb peripheral arterial disease

**DOI:** 10.1186/s12955-017-0760-3

**Published:** 2017-09-22

**Authors:** Anne Sofie F. Larsen, Anne Therese Reiersen, Morten B. Jacobsen, Nils-Einar Kløw, Joakim Nordanstig, Mark Morgan, Jarlis Wesche

**Affiliations:** 1 Department of Radiology, Ostfold Hospital Trust, PB300, 1714 Grålum, Norway; 2 Department of vascular surgery, Ostfold Hospital Trust, Grålum, Norway; 3 Department of internal medicine, Ostfold Hospital Trust, Grålum, Norway; 4 Institute of Clinical Medicine, Faculty of Medicine, University of Oslo, Oslo, Norway; 5 Department of Radiology, Oslo University Hospital, Oslo, Norway; 6 Department of Hybrid and Interventional Surgery, Sahlgrenska University Hospital, Gothenburg, Sweden; 7 Tauranga Public Hospital, Cameron Road, Tauranga, New Zealand; 8 Department of Vascular and Thoracic Surgery, Akershus University Hospital, Lørenskog, Norway

**Keywords:** Peripheral arterial disease, Quality of life, Patient reported outcome measures, Intermittent claudication, Endovascular procedures, Vascular surgical procedures

## Abstract

**Background:**

The VascuQoL-6 (VQ-6) health-related quality of life questionnaire, a short version of the disease-specific VascuQoL-25, was developed for clinical practice and use in vascular registries. The study purpose was to evaluate the validity and reliability of VQ-6.

**Methods:**

VQ-6 was translated to Norwegian with linguistic validation and face value evaluation, and consecutive patients with intermittent claudication (IC) or critical limb ischemia (CLI) were included. All patients completed VQ-6 and Short Form-36 (SF-36), and were evaluated with ankle-brachial index (ABI) measurement pre- and post-exercise, a constant load treadmill test and clinical consultation at baseline and after 4 weeks. Correlation analysis, change statistics and receiver operator characteristics (ROC) curves were used to evaluate reliability, validity and responsiveness to change.

**Results:**

One hundred seventy-one patients with peripheral arterial disease (PAD) were included, 70 (41%) female. 147 (86%) of the patients suffered from IC. The reliability of VQ-6 was good, Cronbachs-α 0.82. The ability of VQ-6 to differentiate between IC and CLI was good, area under the curve (AUC) 0.754. There was good correlation between SF-36 physical domains and component scores and VQ-6 score (*r* = 0.55–0.62) and excellent responsiveness to change after treatment, standard response mean (SRM) 1.12. The clinical anchors of ABI at rest, treadmill walking performance and Fontaine class improvement were less responsive to change than VQ-6, SF-36 and the vascular surgeon’s evaluation.

**Conclusions:**

VQ-6 is reliable and valid, and can be used to evaluate PAD treatment in clinical practice and in vascular registries. Further research is necessary to determine the clinically important change over time.

**Trial registration:**

ISRCTN14846962 (retrospectively registered).

## Background

Patient reported outcome measures (PROM) and patient reported experience measures (PREM) are increasingly important in the evaluation of health care quality. In quality registries, such measures can add important information to the outcome evaluation.

PROMs are usually based on questionnaires [1]. For patients with peripheral arterial disease (PAD) the most used questionnaires assess health-related quality of life (QoL) and functional impairment [2, 3]. Both generic QoL instruments and disease-specific QoL instruments have been used in research for decades, but the use of such measures in clinical practice is still low.

The symptoms of PAD vary from leg claudication (intermittent claudication –IC) to pain at rest and gangrene (critical limb ischemia -CLI), and the natural history of the disease spans from stable disease to the need for vascular reconstruction or leg amputation. In vascular registries, treatment outcome for CLI can be measured through patency of vascularized vessel segments, limb salvage and amputation-free survival. However, the evaluation of outcome after revascularization in low risk patients suffering from IC is more challenging. The severity of claudication symptoms and restrictions in daily life are important factors in determining whether the patient should be offered invasive treatment [4]. To include variables that cover these aspects in vascular registries would enable better outcome assessment, as relief from claudication symptoms and increased walking distance can be considered more important than rare adverse events. The inclusion of PROMs in vascular registries therefore holds promise to improve the patient outcome evaluation.

The Inter-Society Consensus for the Management of Peripheral Arterial Disease (TASC II) [5] recommend use of the physical domains of the generic health-related QoL measure Short Form-36 (SF-36) or the Walking Impairment Questionnaire (WIQ) as patient-based outcome measures for IC in clinical practice. Disease-specific QoL-measures have shown better sensitivity to change following treatment, and a range of different questionnaires for PAD exist [6–8]. Further validation studies of existing PROMs for PAD have been requested [7].

The VascuQoL is a questionnaire developed in the UK for research purposes in 2001 [9]. The original version has 25 items. Validation of VascuQoL-25 (VQ-25) has been performed using selected domains of SF-36 [10], all domains of SF-36 [11], as well as all subscale and component scores of SF-36. The short version, VascuQoL-6 (VQ-6), was developed based on the psychometric properties of VQ-25 in Sweden [12, 13]. This short questionnaire is intended to overcome the reluctance to use QoL-measures in clinical practice by being easy to administer and quick to complete. It also gives a summary measure, useful as an index, and applicable in vascular registries. The measure is recently introduced in the Swedish vascular registry (Swedvasc -http://www.ucr.uu.se/swedvasc/), but VQ-6 has not been validated in a separate study.

The process of validation for a health-related QoL measure is not a simple or finite task, but requires a continuum of evidence based on a series of investigations to assess meaning and usefulness [14]. Validation through an anchor-based approach, where the measure is compared to generic QoL tools and clinical measures is applicable for PAD. As VQ-6 is intended for use in clinical practice, the validation should be performed in this setting. There is a need to translate clinically significant improvement, or deterioration, into points of change for the individual patient.

### Purpose

The aim of this study was to evaluate the validity, reliability and responsiveness of VQ-6, a disease-specific QoL measurement for use in clinical practice and vascular registries.

## Methods

### Inclusion and exclusion

Consecutive patients with new referral for evaluation of peripheral arterial disease (IC or CLI) at the vascular surgery department at two different hospitals (H1 and H2) were invited to participate based on the information given by the referring physician. The inclusion period ran from August 2014 to August 2015. Patients received written information about the study and the two questionnaires, VQ-6 and SF-36, by post before the scheduled appointment at the outpatient clinic. If the patients failed to bring the questionnaires or were admitted acutely, they were invited to participate at site and completed the questionnaires before further investigations. The returned questionnaires were not available to the treating physician.

**Fig. 1:**
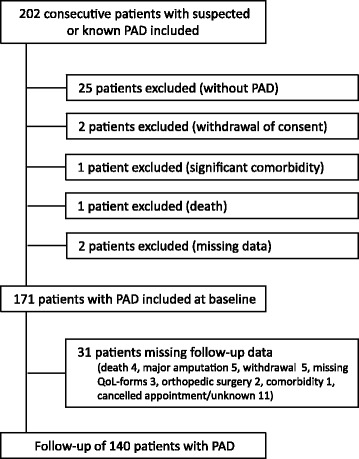
Inclusion and exclusion of patients in the VascuQoL-6 study. PAD –Peripheral arterial disease, IC-intermittent claudication, CLI –critical limb ischemia

If the consulting vascular surgeon ruled out symptomatic PAD, the patients were excluded. For the patients who died, underwent major amputation or major non-vascular surgery, or moved out of the hospital region, only baseline data was available. Patients also had the opportunity to withdraw at all times. Inclusion, exclusion and follow-up of patients are shown in Fig. 1.

### Work up

In the vascular laboratory, arterial pressures were measured with a hand held Doppler device (H1: Model 811-BTS (9.5 MHz) Parks medical electronics INC, Aloha, Oregon, USA, H2: Model 811-BTS (8.2 MHz) Parks medical electronics INC, Aloha, Oregon, USA), and the ankle-brachial index (ABI) was calculated. Patients with claudication were tested on a constant load treadmill with a speed of 2.5 km/h and no inclination (H1: Abilica X-fit 1, Myrnasport, Norway, H2: Woodway PPS Med, Woodway GmbH, D79576 Weil am Rhein, Germany). A post-exercise ABI drop exceeding 0.1 was regarded significant. Intermittent claudication distance (ICD) and maximum walking distance (MWD) were registered in meters. There is a ceiling effect at 416 m, as the test was terminated after 10 min for all patients.

Risk factors, comorbidity, medication and Fontaine classification [15] were registered by the vascular surgeon during the clinical consultation.

### Follow-up

All patients were scheduled for a new consultation with completion of questionnaires, arterial pressure measurements, treadmill-test and clinical evaluation. Patients receiving conservative treatment (information about the disease, the value of walking exercise and medical treatment) and patients referred for supervised exercise therapy were followed up after 4 weeks. The referral algorithm for imaging and invasive treatment was unaltered from usual practice. If results from imaging indicated a conservative approach, the patients had their follow-up as soon as possible. Patients referred for invasive treatment (endovascular or surgical) underwent follow-up 4 weeks after the invasive procedure.

### Translation and adaption of VascuQoL-6

The VQ-6 was translated to Norwegian from the Swedish version using the method of linguistic adaption and validation described by the MAPI institute [16], including a forward and backward translation to English. Face value of the questionnaire was tested by five patients and five experienced vascular surgeons, by interviews, answering three questions: Are the questions easy to understand? Do you find them relevant for your condition/your patients? Do you have any suggestions for alterations (language/missing items, scaling, etc.)? This evaluation resulted in a slight adjustment of wording. The final version was approved by the original developers.

Each of the six items scores from one to four, sum score range is from six to 24, and a higher score indicates better health.

### SF-36

**Fig. 2:**
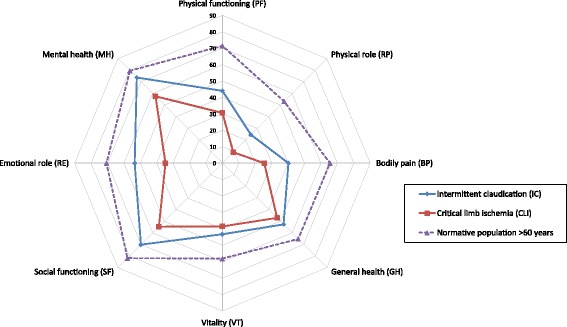
SF-36 subscale mean scores at baseline (*n* = 171). Patients suffering from intermittent claudication (IC) and critical limb ischemia (CLI). For comparison Norwegian norm population aged 60 years and over (Loge 1998). PF - physical functioning, RP - physical role limitations, BP - bodily pain, GH - general health, VT - vitality, SF - social functioning, RE - emotional role limitations, MH - mental health

SF-36 (version 1) was chosen as generic QoL anchor, as this questionnaire has been used in prior validation of VascuQoL-25 [10, 11, 13, 17] and as QoL measure in a range of PAD studies [18–21]. This enabled comparison with earlier research. Subscale (PF –physical functioning, RP –physical role, BP –bodily pain, GH –general health, VT –vitality, SF -social functioning, RE –emotional role, MH –mental health) and component summary scoring (PCS –physical component score, MCS –mental component score) was performed using Qualimetric Health Outcomes Scoring Software 4.0, using the original scoring method [22]. This software uses the 1998 US norm population for calculation of component summary scores, as US norm has been recommended for western countries [23]. Data from the Norwegian norm population from 1998 [24] was used in Fig. 2 for illustration purposes.

The subscale scores of SF-36 range from 0 to 100, and the highest score indicates no health-related reduction of QoL. The component summary scores relates to the normative population (mean 50, SD 10), and a score lower than 50 indicates lower QoL than the normative population.

### Power analysis

The power analysis was conducted for the responsivity to change after treatment. An evaluation of the practice at H1 showed that one third of patients referred with IC and most patients with CLI would receive invasive treatment. Registrations of invasive treatment from the Norwegian vascular registry (NORKAR- www.norkar.no) showed an approx. 15–18% proportion of CLI at H1. Earlier studies have shown 20–32% improvement in VascuQoL-25 summary score after invasive treatment [21, 25]. A 25% improvement would translate to a four point improvement in VQ-6 summary score and the strength evaluation was done with the aim to detect four points improvement for at least 30% of the invasive treatment group and less than 5% of the conservative treatment group.

### Statistic methods

All statistics were calculated using Statistical Package for the Social Sciences version 21 (IBM/SPSS Inc., Armonk, NY, USA).

#### Reliability (internal consistency)

Cronbach’s alpha was calculated to evaluate the internal consistency of the VQ-6. A reliable health-related QoL-measure should center between 0.7 and 0.9 for group comparisons [26]. The intraclass correlation coefficient (ICC, two-way mixed model with 95% confidence interval, absolute agreement of average measures) was calculated to test-retest reliability for patients in the conservative treatment group, as a priori no health change was expected for this group in the short time span of 4 weeks.

#### Validity (accuracy)

Content validity was assessed through face value evaluation and through the earlier development processes [9, 12].

The construct validity (cross-sectional construct validity, sensitivity to differences) was assessed through the instrument’s ability to discriminate between patients suffering from IC and CLI, using Receiver operating characteristic (ROC) curve analysis, with the Fontaine classification as a basis. The area under the ROC curve (AUC) benchmarks were > 0.8 (excellent), 0.7–0.8 (fair) and < 0.7 (poor).

Criterion validation was performed through exploration of correlations between SF-36 domain and summary scores and VQ-6 sum scores, using Pearson product-moment correlation coefficient for normally distributed variables and Spearman’s rank correlation coefficient as a non-parametric alternative. Interpretation of correlation coefficients was done using Cohen’s criteria: small 0.1–0.29, medium 0.3–0.49 and large 0.5–1.0.

Standard and hierarchical linear multiple regressions were used to model the relationship between the questionnaires while controlling for other variables, after checking for violation of assumptions (statistical power, multicollinearity, singularity, outliers and normality).

#### Responsiveness

Change analysis was done using t-test for normally distributed continuous data, Wilcoxon signed rank test for non-parametric continuous data and McNemar’s test for dichotomous data.

ROC curves were calculated for comparison with clinical anchors of change.

Standardized response means (mean change divided by the standard deviation of the change) of SF-36 domains and component summary scores and all items and summary score of VQ-6 were calculated to evaluate responsiveness [27].

To determine the clinically important change in VQ-6, the minimally important difference (MID) was calculated using the distribution based method with 0.5 SD [28] as well as anchored in a clinical evaluation by the vascular surgeon [29].

#### Missing data

Analysis was performed using all available data at baseline and during follow-up. If only one item was missing for VQ-6 (3 patients) we did a single imputation using the median of the item score for all patients. One patient missed one item after treatment, and the sum score of VQ6 was omitted from analysis. For SF-36, imputation was done using the scoring software (Qualimetric Health Outcomes Scoring Software 4.0). The mean subscale score is used if the patient has answered more than half of the items in the domain.

## Results

One hundred seventy-one patients were included, 41% female. Some 31% had a previous history of evaluation or treatment for PAD. Patient characteristics are given in Table 1. Of the patients, 86% were claudicants (Fontaine IIA/B) and 14% suffered from critical limb ischemia (Fontaine III and IV). A total of 83% participated in a treadmill test. Arterial pressures, treadmill walking capacity and QoL-summary scores at baseline and follow up is shown in Table 2. SF-36 profiles at baseline are shown in Fig. 2.

**Table 1:** Patients characteristic (*n* = 171)

	N (eligible for analysis)	Percent	Median (range)
Age	171		
Male		59.1%	70 (47–89)
Female		40.9%	71 (44–89)
BMI	162		26.6 (16.4–41.2)
Smoking^a^	169	60.9%	
Diabetes	171	21.1%	
Impaired renal function	137		
eGFR < 60		21.2%	
eGFR < 45		8.8%	
Anti-hypertensive treatment	151	74.8%	
Cerebrovascular disease	171	15.8%	
Cardiovascular disease	171	39.2%	
Chronic obstructive pulmonary disease	171	18.1%	
Other comorbidity	171	14.6%	
Work status	168		
Paid work		15.5%	
Sick leave or disability pension		18.4%	
Retired or unpaid work		66.1%	

^a^Smoking or previous smoking within 5 years

**Table 2:** Quality of life summary scores, arterial pressure indices and walking capacity at baseline and follow-up

	All participants (*n* = 171)	Conservative treatment (*n* = 68)			Invasive treatment (*n* = 73)			No follow-up (*n* = 30)
	Baseline	Baseline	Follow-up		Baseline	Follow-up		Baseline
	Mean (95% CI)	Mean (95% CI)	Mean (95% CI)	*p*	Mean (95% CI)	Mean (95% CI)	*p*	Mean (95% CI)
VQ-6 sum	12.7 (12.2–13.3)	13.6 (12.8–14.4)	15.2 (14.3–16.1)	*0.001* ^*b*^	12.1 (11.4–12.8)	19.6 (15.8–18.0)	*0.001* ^*b*^	12.4 (10.7–14.0)
SF-36 PCS	33 (32–34)	35 (33–36)	38 (36–40)	*0.001* ^*b*^	32 (30–33)	40 (37–42)	*0.001* ^*b*^	32 (30–35)
SF-36 MCS	48 (47–50)	51 (48–53)	48 (45–51)	*0.470* ^*b*^	48 (45–51)	48 (46–51)	*0.780* ^*b*^	45 (40–50)
ABI^a^	0.62 (0.59–0.65)	0.65 (0.62–0.69)	0.67 (0.63–0.71)	*0.394* ^*b*^	0.57 (0.52–0.62)	0.76 (0.71–0.82)	*0.001* ^*b*^	0.64 (0.57–0.72)
ABI pe^a^	0.47 (0.43–0.51)	0.55 (0.50–0.60)	0.53 (0.47–0.59)	*0.597* ^*b*^	0.36 (0.29–0.43)	0.67 (0.60–0.75)	*0.001* ^*b*^	0.54 (0.42–0.67)
	Median (IQ)	Median (IQ)	Median (IQ)		Median (IQ)	Median (IQ)		Median (IQ)
ICD	87 (46–133)	100 (55–159)	107 (10–600)	*0.036* ^*c*^	62 (40–100)	110 (0–295)	*0.001* ^*c*^	110 (53–145)
MWD	400 (164–410)	410 (260–418)	410 (30–650)	*0.066* ^*c*^	233 (110–400)	403 (30–570)	*0.001* ^*c*^	410 (240–420)

VQ-6 –Vascular Quality of Life Questionnarie-6, SF-36 –Short Form-36, PCS –Physical component summary score, MCS –Mental component summary score, ABI -ankle-brachial index, pe –postexercise, ICD –intermittent claudication distance, MWD –maximum walking distance, IQ –interquartile range (25th–75th percentile)

^a^Symptomatic leg

^b^Two-tailed t-test for normally distributed data, *p* < 0.05 was regarded significant

^c^Wilcoxon signed rank test for non-normally distributed data, *p* < 0.05 was regarded significant

### Reliability

As determined by Cronbach’s alpha coefficient calculations at baseline (*n* = 171), the VQ-6 demonstrated good internal consistency (alpha = 0.82).

In the conservative treatment group (*n* = 68, IC/CLI: 68/0), 19 patients (28%) improved four points or more in VQ-6 summary score after 4 weeks. There was a statistically significant improvement in VQ-6 summary score and SF-36 PCS mean. The SF-36 MCS, ABI at rest, ABI post-exercise and MWD was unchanged from baseline (Table 2). The proportion of patients able to complete the treadmill test (MWD > 400 m) increased, from 69% to 80%. ICC for VQ-6 summary score at baseline and follow up was 0.66 for the conservative treatment group, indicating good reliability for IC.

### Validity

#### Construct validity

**Fig. 3:**
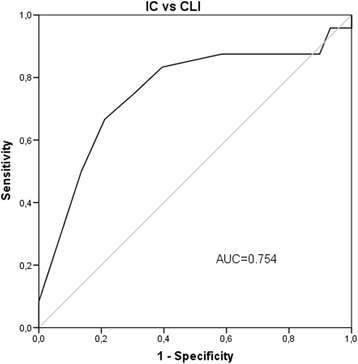
Sensitivity to disease severity. Receiver operator characteristics (ROC) curve of VQ6 summary score at baseline (*n* = 171)

The ability of VQ-6 to differentiate between IC and CLI at baseline are shown in Fig. 3. From the ROC curve, a discriminative cut -off point (IC versus CLI) of 11 is suggested. The test’s sensitivity to detect CLI if the score is 11 or lower is 0.75, with a specificity of 0.69. At baseline, two patients (1.2%) scored at the lowest possible VQ-6 level, and none at the highest level, suggestive of limited problems with floor and ceiling effects.

#### Correlation

Correlations between VQ-6 and SF-36 at baseline are given in Table 3. There was a strong positive correlation between the VQ-6 summary score and the PCS of SF-36 (rho = 0.55), and a fair correlation between VQ-6 summary score and the MCS (rho = 0.49). The correlation between the domains of physical functioning, physical role and bodily pain and VQ-6 summary score was even stronger (PF: rho = 0.62, RP: rho = 0.56, BP: rho = 0.59).

**Table 3:** Correlation coefficients (Spearman’s rho) for SF-36v1 domains and individual items of VascuQoL-6 at baseline (*n* = 171)

	Item 1	Item 2	Item 3	Item 4	Item 5	Item 6	
SF-36	*Activity*	*Symptoms (weakness)*	*Activity (walking)*	*Emotional (concern)*	*Social activities*	*Pain*	VQ6 SUM
Physical functioning	0,536	0.520	0.489	0.308	0.586	0.355	0.643
Physical role	0.527	0.473	0.334	0.276	0.458	0.375	0.563
Bodily pain	0.464	0.492	0.339	0.328	0.479	0.441	0.586
General health	0.314	0.278	0.279	0.307	0.322	0.126*	0.388
Vitality	0.370	0.413	0.218	0.310	0.403	0.232**	0.461
Social functioning	0.422	0.340	0.376	0.408	0.528	0.327	0.566
Emotional role	0.386	0.336	0.245	0.322	0.449	0.333	0.493
Mental health	0.360	0.296	0.276	0.412	0.435	0.242	0.473
Physical component score	0.497	0.475	0.422	0.237**	0.447	0.319	0.555
Mental component score	0.374	0.319	0.252	0.401	0.469	0.289	0.502

*p* ≤ 0.01, *p = 0.104. **p = 0.002

#### Regression

Hierarchical multiple linear regression was used to assess the ability of the generic health related QoL measure (SF-36), ABI at rest and the patients’ ability to participate in a treadmill test to predict the disease-specific QoL (VQ-6 summary score) at baseline, after controlling for age, gender, BMI, smoking status and comorbidity. The total variance explained by the model was 59%. The factors controlled for could explain 4.7% of the variance in VQ-6 summary score. The SF-36 domains, ABI at rest and ability to perform on a tread-mill explained 54.1% of the variance after controlling for age, gender, BMI, smoking and comorbidity (r square change 0.541, *p* < 0.001). In the final model, only the contribution of PF and BP were statistically significant (beta = 0.245 and 0.263, *p* < 0.01).

A separate regression model was used to assess only the patients able to perform the walking test (*n* = 142). This hierarchical regression model included the SF-36, ABI at rest, an ABI drop > 0.1 and MWD, controlling for age, gender, BMI, smoking and comorbidity. The total variance explained by the model was 58.2%. The factors controlled for could explain 3.7% of the variance in VQ-6 summary score. The SF-36, ABI at rest, ABI drop and MWD explained 54.5% of the variance after controlling for age, gender, BMI, smoking and comorbidity (r square change 0.545, *p* < 0.001).

#### Responsiveness to change

In the invasive group (*n* = 73, IC/CLI: 60/13), 41 patients (56%) improved four points or more in VQ-6 summary score. There was a statistically significant improvement in VQ-6 summary score and all domains except RE for the invasive group, compared to only two domains for the conservative group (PF and BP).

**Fig. 4:**
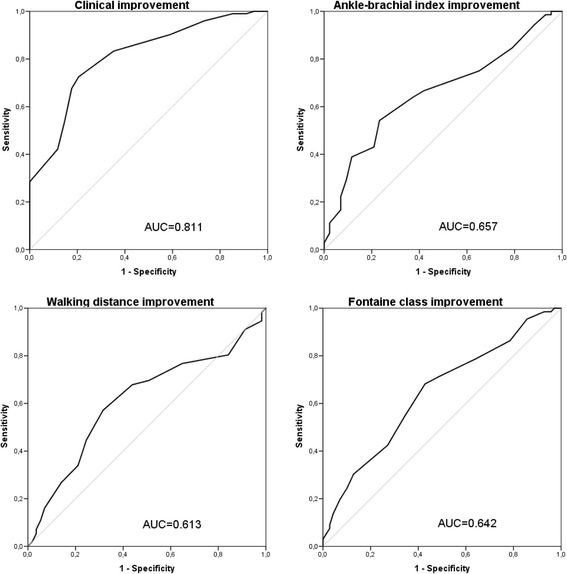
Responsiveness to change. Receiver operator characteristics (ROC) curve of VQ6 summary score change compared to clinical improvement as evaluated by the vascular surgeon, improvement of ABI at rest > 0.1, improvement in treadmill walking distance of more than 50% and improvement in Fontaine class

The responsiveness to change for VQ-6 anchored in clinical evaluation and clinical measures is illustrated in Fig. 4. The ROC curves of VQ-6 summary score is plotted against the dichotomous variables; clinical improvement as evaluated by the vascular surgeon, improvement of ABI at rest of more than 0.1, improvement of MWD of at least 50% and improvement in Fontaine classification.

**Fig. 5:**
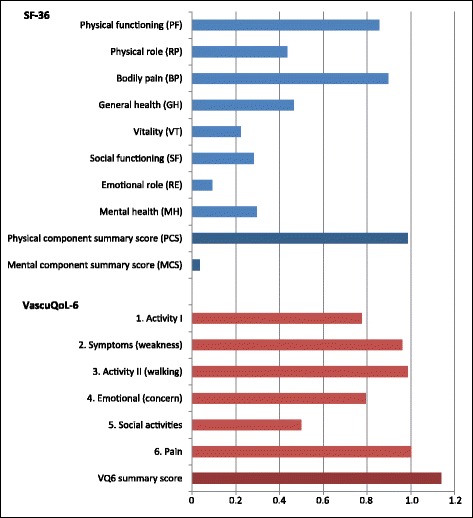
Responsiveness to change. Standard response mean (SRM) of SF-36, domains and component summary scores, and VQ6, all items and summary score

Standardized response means of SF-36 domains and component summary scores and all items and summary score of VQ-6 are shown in Fig. 5. Using Cohen’s criteria of effect size, there is a moderate to large effect for all VQ-6 items, and a large effect size for VQ-6 summary score (1.13). Using the criteria of 0.5 SD of baseline VQ-6 summary score for MID, the value is 1.725, which translates to 2 points. For patients where the vascular surgeon evaluated the symptoms to be unchanged, the mean change in VQ-6 summary score was −0.27 (95% CI: -1.38–0.82). This suggests a MID for improvement of 0.82 points from baseline, and for deterioration of −1.38, using upper and lower confidence intervals as suggestive for MID.

## Discussion

In this study of the psychometric properties for the health-related quality of life questionnaire VascuQoL-6, a good internal consistency, a good correlation with the physical domains and physical component summary score of SF-36 and an excellent responsiveness to clinical change were demonstrated. The clinical anchors of ABI, post-exercise ABI and walking capacity also improved significantly after treatment. The comparison of the VQ-6 scores with the clinical anchors of ABI at rest and a fixed speed treadmill test illustrate the well-known shortcomings of these outcome measures [30, 31]. The correlations between the two QoL measures are good, but lower than in the original development process of VQ-6. Both patient samples include patients with IC and CLI, with a higher proportion of CLI (36% versus 14%) among the patients in the development process. In the study by Nordanstig et al., the Swedish normative population was used when calculating PCS and MCS, and the correlation for these parameters is thus not directly comparable to our results.

Our patients were unselected, but the regression analysis does not point towards age, obesity or comorbidity as important factors in explaining the variance in the PROMs. Overall, the results from the regression model suggest that the QoL measures constitute important individual outcome measures not covered by physical measurements of ABI and walking capacity. This was also indicated by Mazari et al. in their comparison of the SF-36 and the VQ-25 to walking distances and arterial pressure measurements after percutaneous transluminal angioplasty, where they found a moderate correlation with treadmill distance and only a weak correlation with ABI at rest and after exercise [21].

We observed more than four points of change for two thirds of the patients in the invasive group and in one third of the conservative treatment group. The strength evaluation was done expecting a change of four points for at least 30% of the invasively treated patients, while we expected a smaller change for patients with conservative treatment. In the short time span of 4 weeks, minimal physical benefit from exercise or optimized medical treatment could be expected. The severity of disease was lower in the conservative treatment group, where symptoms of claudication were predominant. This means that we had a large effect of information and reassurance given to the patient. Some of the improvement could also be the result of response shift (the patient adapting to the diagnosis). To evaluate reliability through test and retest, ICC was calculated for the conservative group, and the result of 0.66 indicates good reliability, but may be underestimated in this study, the observed improvement in QoL scores for the conservative treatment group taken in account. As the conservative group was restricted to claudicants, reliability has not been tested with ICC for patients with CLI.

SF-36 is a profile, while VQ-6 gives an index. This complicates the comparison of these instruments. The component summary scores of SF-36 are less sensitive to changes affecting only a few domains, as for patients with PAD. Low scores in physical domains will inflate the MCS and visa versa [32].

Since the two instruments were administered together, external factors not specific to the disease (patients feel sad, tired, hungry etc.) could influence the answers to both questionnaires, and lead to a higher correlation than the underlying traits account for [14]. This effect is not possible to quantify. Most patients received information about the study and filled in their questionnaires at home, but a possible at site recruitment bias can have occurred. For patients with CLI and acute admission, at site recruitment was the only option to include these patients.

VascuQoL is intended to cover the spectrum of severity of PAD. Patients with CLI will probably have a lower score due to pain and concern, while patients with IC have more restrictions in activity. As the short form (VQ-6) contains two items about activity (activity and walking), the relationship between VQ-6 summary score and clinical improvement may be greater for patients with IC.

Clinically significant improvement or deterioration needs to be translated into points of change in results from the questionnaire in the individual patient. The MID indicates the lowest change in score that can be interpreted as improvement or deterioration relevant to the patient. For VQ-25 the MID has been discussed in recent articles by Conijn [33, 34]. As we anchored MID calculation in an evaluation of symptom change by the vascular surgeon, there is a possible bias towards improvement after treatment. Based on our analysis, combined with earlier research concerning correlation between VQ-6 and VQ-25 [12] and change in VQ-25 score [21, 25], we would recommend two points of change as indicative and four points as a certain change in either direction for the individual patient after treatment. This is probably a conservative recommendation, but further research in larger patient samples is needed to establish how many points of change constitutes the “true” change.

## Conclusions

VQ-6 is a reliable and valid instrument for evaluation of QoL in patients suffering from PAD in clinical practice, and the summary score can be used in group comparisons, for instance in vascular registries. PROMs constitute an important individual outcome measure not covered by physical measurements of ABI and walking capacity.

## Abbreviations

ABI: Ankle-brachial index
BP: Bodily pain, Short Form-36
CLI: Critical limb ischemia
GH: General health, Short Form-36
IC: Intermittent claudication
ICD: Intermittent claudication distance
MCS: Mental component score, Short Form-36
MH: Mental health, Short Form-36
MID: Minimally important difference
MWD: Maximum walking distance
PAD: Peripheral arterial disease
PCS: Physical component score, Short Form-36
PF: Physical functioning, Short Form-36
PREM: Patient reported experience measures
PROM: Patient reported outcome measures
QoL: Quality of life
RE: Emotional role, Short Form-36
ROC: Receiver operator curve
RP: Physical role, Short Form-36
SF: Social functioning, Short Form-36
SF-36: Short Form-36
VascuQoL: Vascular quality of life questionnaire
VQ-25: Vascular quality of life questionnaire-25
VQ-6: Vascular quality of life questionnaire-6
VT: Vitality, short form-36
WIQ: Walking impairment questionnaire
